# Investigating the Bias in Orthopaedic Patient-reported Outcome Measures by Mode of Administration: A Meta-analysis

**DOI:** 10.5435/JAAOSGlobal-D-20-00194

**Published:** 2020-12-04

**Authors:** Jonathan Acosta, Peter Tang, Steven Regal, Sam Akhavan, Alan Reynolds, Rebecca Schorr, Jon E. Hammarstedt

**Affiliations:** From the Department of Orthopaedic Surgery, Allegheny General Hospital (Dr. Acosta, Dr. Tang, Dr. Regal, Dr. Akhavan, Dr. Reynolds, and Dr. Hammarstedt); and the Highmark Health Data Science R&D (Ms. Schorr), Pittsburgh, Pennsylvania.

## Abstract

**Background:**

Patient-reported outcome measures (PROMs) are critical and frequently used to assess clinical outcomes to support medical decision-making.

**Questions/Purpose:**

The purpose of this meta-analysis was to compare differences in the modes of administration of PROMs within the field of orthopaedics to determine their impact on clinical outcome assessment.

**Patients and Methods:**

The PubMed database was used to conduct a review of literature from 1990 to 2018 with the Preferred Reporting Items for Systematic Reviews and Meta-Analyses protocol. All articles comparing PROMs for orthopaedic procedures were included and classified by the mode of administration. Each specific survey was standardized to a scale of 0 to 100, and a repeated random effectsmodel meta-analysis was conducted to determine the mean effect of each mode of survey.

**Results:**

Eighteen studies were initially included in the study, with 10 ultimately used in the meta-analysis that encompassed 2384 separate patient survey encounters. Six of these studies demonstrated a statistically notable difference in PROM scores by mode of administration. The meta-analysis found that the standardized mean effect size for telephone-based surveys on a 100-point scale was 71.7 (SE 5.0) that was significantly higher (P , 0.0001) than survey scores obtained via online/tech based (65.3 [SE 0.70]) or self-administered/paper surveys (61.2 [SE 0.70]).

**Conclusions:**

Overall, this study demonstrated that a documented difference exists in PROM quality depending on the mode of administration. PROM scores obtained via telephone (71.7) are 8.9% higher than scores obtained online (65.3, P , 0.0001), and 13.8% higher than scores obtained via self-administered on paper (61.8, P , 0.0001). Few studies have quantified statistically notable differences between PROM scores based solely on the mode of acquisition in orthopaedic

Patient-reported outcome measures (PROMs) are health outcomes reported directly by the patients. They are standardized, validated tools and instruments used to measure patient perception of functional outcomes and health status and have the ability to detect underlying change in physical status. They are frequently used by clinicians and researchers to assess clinical outcomes to support decision-making.^1,2^ Data generated via PROMs influences future research and health policy to guide and improve on healthcare delivery.^3,4^ Collection of PROMs has become increasingly common because healthcare systems focus on value-based care, which affects reimbursement.^4,5^ PROMs are able to capture data about the patient's mental, physical, and emotional status including pain level, activity level, and functional status at multiple time points along the patient's injury or disease episode.^2,6^ Furthermore, PROMs may be obtained in a variety of modalities including in-person surveys, phone calls, online/technology-based surveys, and self-administered/paper surveys. The specific mode of PROM collection may be a confounding variable and cause collection bias; however, few studies report their method of collection.^7,8,9^

Researchers have previously investigated the effects of survey mode of administration on PROMs. These studies explore potential biases in fields such as oncology, addiction, and others and have showed that having interviewers present, whether over the phone or in person, can artificially elevate PROM scores up to 15% higher compared with PROM scores done without an interviewer.^10,11,12,13,14,15^ This is known as interview bias. A lack of understanding exists of the PROM administration mode bias within orthopaedic surgery, a field in which PROMs are essential. For instance, the American Board of Orthopaedic Surgery now collects PROM data to help inform decision-making for their board certification process.^16^

The purpose of this meta-analysis was to compare differences in the modes of administration of PROMs within the field of orthopaedics to determine the impact on clinical outcome assessment. We hypothesized that there would be statistically notable higher PROM scores obtained by telephone when compared with other modes of administration.

## Methods

### Search Strategy

**Figure 1:**
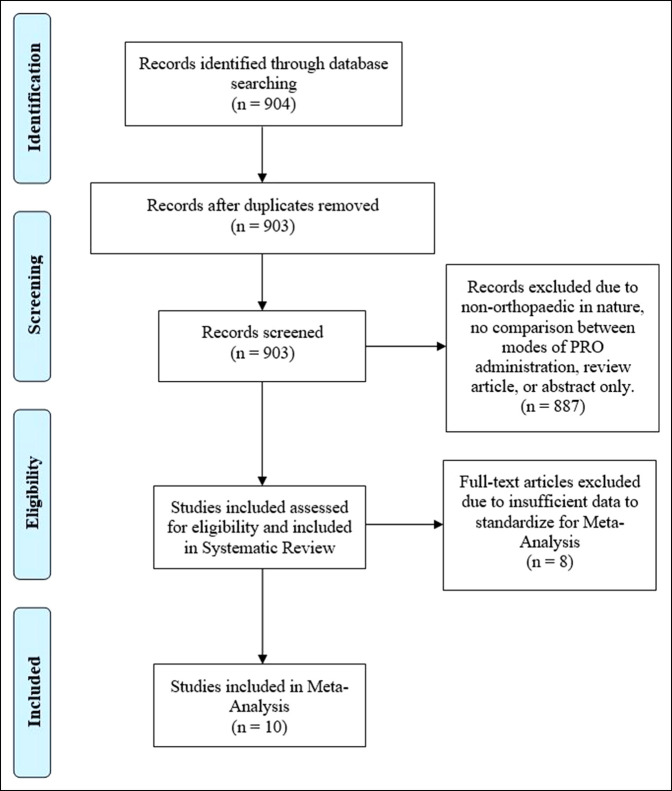
Preferred Reporting Items for systematic Reviews and Meta-Analyses flow diagram demonstrating orthopaedic patient-reported outcome measure comparison studies.

The PubMed database was used to conduct review of the literature from 1990 to 2018 with the Preferred Reporting Items for Systematic Reviews and Meta-Analyses protocol to identify studies that compared the mode of survey administration and patient-reported outcomes. Search terms used in the title, MeSH, and keywords included “Data Collection/Methods,” “Survey and Questionnaires,” “Health Care Survey,” “Patient Reported Outcome Measures,” “Patient Outcome Assessment,” “Musculoskeletal System/surgery,” “Musculoskeletal Disease/Surgery,” “Orthopaedic Procedures,” “Bias,” “Interviews,” “Telephone,” “Postal Service,” and “Electronic Mail”. References from the included articles were also examined for inclusion that may have been missed by the initial literature search. The details of study identification, screening, inclusion, and exclusion can be found in Figure 1.

### Study Selection and Criteria

Study selection for this meta-analysis was determined by two independent reviewers based on the defined selection criteria. Studies were selected for the meta-analysis if they were in the field of orthopaedic surgery, compared the results of PROMs because it pertained to the mode of administration, published in a peer-reviewed journal, and were written in the English language or had translation of text readily available. Studies were excluded if they were not in the field of orthopaedic surgery, had no comparison between modes of PROM administration, were a review article, or were only an abstract.

### Meta-analysis

In total, 18 studies were present in this meta-analysis that included a total of 4408 patient encounters who were involved in investigations of patient-reported outcome data across multiple modes of administration. Eight studies were excluded because of insufficient data. Ten studies (n = 2384) were ultimately included in the meta-analysis. The studies included in the meta-analysis area summarized in Table 1. All involved patients were receiving treatment of an orthopaedic condition. Each study used a validated patient survey specific to the condition being treated and compared the scores noted among the different modalities of collection. Such modalities include online/technology-based, telephone, in-person interview, in-office self-administered/paper survey, and postal survey.

**Table 1:** Summary Table of the 10 Studies Included in the Review and Meta-analysis Including Title, First Author, Journal, Year of Publication, Procedure, PRO Measured, and Method of Administration

Title	1st Author	Year	Procedure	PRO	Online/Tech Based			Telephone			In-Person Interview			Self-Administered/Paper			Postal		
					Score	n	f/u time (m)	Score	n	f/u time (m)	Score	n	f/u time (m)	Score	n	f/u time (m)	Score	n	f/u time (m)
Survey mode influence on patient-reported outcome scores in orthopaedic surgery: telephone results may be positively biased	Hammarstedt	2015	Arthoscopic labrum repair	mHHS	80.4	138	24	86.9	145	24				80.6	102	24			
				HOS-ADLS	81.5	138	24	89.1	145	24				82.3	102	24			
				HOS-SSS	66.1	138	24	75.6	145	24				65.6	102	24			
				NAHS	80.1	138	24	84.5	145	24				80.3	102	24			
				VAS	2.8	138	24	3.1	145	24				2.8	102	24			
A comparative study of telephone versus onsite completion of the WOMAC 3.0 osteoarthritis index	Bellamy	2002	Knee osteoarthritis	WOMAC pain				8.61	50					8.69	50				
				WOMAC stiffness				4.42	50					4.54	50				
				WOMAC function				31.41	50					32.18	50				
				WOMAC total				44.43	50					45.41	50				
Does the mode of data collection change results in a subjective knee score?	Hoher	1997	ACL surgery	Lysholm score							92.2	61	12	89.3	61	12			
Patients respond similarly to paper and electronic versions of the WOMAC and SF-12 following total joint arthroplasty	Marsh	2014	THA/TKA	WOMAC	21.72	53	12							21.76	53	12			
				SF12PCS	44.5	53	12							42.7	53	12			
				SF12MCS	50.27	53	12							51.44	53	12			
Mail versus telephone administration of the oxford knee and hip scores	Messih	2014	THA/TKA	OKS				15.79	85								15.58	85	
				OHS				13.54	61								12.34	61	
Is it too early to move to full electronic PROM data collection? A randomized controlled trial comparing PROM's after hallux valgus captured by e-mail, traditional mail, and telephone	Palmen	2015	Hallux valgus surgery	FFI	26.6	24		21.7	25								28.3	24	
				EQ5D index	6	24		6.05	25								6.09	24	
				EQ VAS	86.3	24		83	25								80	24	
Are patient-reported outcome measures biased by the method of follow-up? Evaluating paper-based and digital follow-up after lumbar fusion surgery	Schroder	2018	Lumbar fusion	ODI	16.82	40	24							17.48	40	24			
				NRS-BP	3.72	40	24							2.78	40	24			
				NRS-LP	2.9	40	24							2.65	40	24			
Patient-reported outcome measures: How do digital tablets stack up to paper forms? A randomized, controlled study	Shah	2016	Upper extremity, spine, arthroplasty services	EQ-5d	0.664	258								0.699	225				
				VAS	62.5	258								71.6	225				
				ODI	41.8	258								36.6	225				
				NDI	42.8	258								32.4	225				
				HOOS	51.6	258								46.2	225				
				KOOS	40.4	258								38.8	225				
				QuickDASH	40.5	258								32.8	225				
Comparison of paper and computer-based questionnaire modes for measuring health outcomes in patients undergoing total hip arthroplasty	Shervin	2011	THA	Harris hip score (touch screen, web)	76, 75.8	57								74.8					
				HHS pain score	30.7, 31.1	57								30.5	57				
				HHS function	37.5, 37.5	57								37.6	57				
				HHS range of motion	3.7, 3.5	57								3.7	57				
				WOMAC	10.10	57								13	57				
				WOMAC pain	2, 1	57								1	57				
				WOMAC function	6.7	57								11	57				
				SF36	79, 78	57								78	57				
				SF36MCS	85, 85	57								86	57				
				SF36PCS	66, 64	57								60	57				
				EQ5D index	0.73, 75	57								0.73	57				
				EQ5D pain	2.2	57								2	57				
				EQ5D function	4.4	57								4	57				
				UCLA activity score	6.6	57								6	57				
Comparison of paper and electronic surveys for measuring patient-reported outcomes after anterior cruciate ligament reconstruction	Bojcic	2014	ACL reconstruction	KOOS: Function in daily living score	92.7	101	12										93.6	127	12
				KOOS: Pain score	86.9	101	12										87.5	127	12
				KOOS: Quality of life score	62.9	101	12										61.3	127	12
				KOOS: Symptoms score	80.7	101	12										65.8	127	12
				KOOS: Function in sport and recreation score	70.9	101	12										72.7	127	12
				KOOS: Function in daily living score	91.2	137	24										90.9	121	24
				KOOS: Pain score	85.3	137	24										85.6	121	24
				KOOS: Quality of life score	63.8	137	24										65.3	121	24
				KOOS: Symptoms score	79.6	137	24										65.3	121	24
				KOOS: Function in sport and recreation score	73.2	137	24										72.9	121	24
				KOOS: Function in daily living score	93.7	63	60										94	80	60
				KOOS: Pain score	87.3	63	60										89.6	80	60
				KOOS: Quality of life score	71.7	63	60										77.6	80	60
				KOOS: Symptoms score	82.3	63	60										67.6	80	60
				KOOS: Function in sport and recreation score	75.6	63	60										79.1	80	60
				KOOS: Function in daily living score	92.2	301	Total combined										92.7	328	Total combined
				KOOS: Pain score	86.3	301	Total combined										87.3	328	Total combined
				KOOS: Quality of life score	65.1	301	Total combined										66.7	328	Total combined
				KOOS: Symptoms score	80.6	301	Total combined										66	328	Total combined
				KOOS: Function in sport and recreation score	72.9	301	Total combined										74.3	328	Total combined

PROM = Patient-reported outcome measure; PRO = Patient-reported outcome; THA = total hip arthroplasty; TKA = total knee arthroplasty; KOOS = Knee Injury and Osteoarthritis Outcome Score; HHS = Harris Hip Score; HOS-ADLS = Hip Outcome Score – Activities of Daily Living; HOS-SSS = Hip Outcome Score-Sports Specific Scale); NAHS = Nonarthritic Hip Score; VAS = Visual Analog Scale; WOMAC = Western Ontario and McMaster Universities Osteoarthritis Index; ACL = anterior cruciate ligament; SF = short form; THA = total hip arthroplasty; TKA = total knee arthroplasty; PCS = Physical Health Composite Score; MCS = Mental Health Composite Score; OKS = Oxford Knee Score; OHS = Oxford Hip Score; FFI = Foot Function Index; EQ5D = EuroQoL-D Dimensions; ODI = Oswestry Disability Index; NRS-BP = Numeric Rating Scale-Back Pain; NRS-LP = Numeric Rating Scale-Leg Pain; NDI = Neck Disability Index; HOOS = Hip Disability and Osteoarthritis Outcome Score; QuickDASH = Disabilities of the Arm, Shoulder, and Hand Score; UCLA = University of California Los Angeles Activity Score

PROMs inherently are each scaled differently depending on what outcome is being assessed. Using a linear approach to scale homogenization simplifies interpretation by designating higher scores as more positive clinical outcomes and lower scores as negative outcomes.^17^ This approach assumes equal distance between values. Mean scores were transformed using the percentage scale maximum method allowing for normalization of the data on a scale from 0 to 100. Heterogeneity was assessed using general linear model which hypothesized that the studies come from a homogeneous population, asymptotic covariance matrix, and restricted maximum likelihood. A forest plot was created to visually assess the different studies stratified by the mode of survey. Covariance parameters and covariance ratios were analyzed and graphed to determine the parameter effect of any outliers in the data testing for heterogeneity. Restricted maximum likelihood was conducted to account for the covariance between studies. A repeated random effects model meta-analysis was conducted to determine the mean effect of each mode of survey. This model controlled for heterogeneity because parameters in the model and residuals were held to known values. SAS Enterprise Guide 7.15 HF3 (SAS Institute, Inc) was used to conduct the statistical analysis.

## Results

### Electronic-/Technology-Based Surveys

Two orthopaedic surgery specific studies included in the meta-analysis showed no notable differences between tablet/computer and paper survey scores.^18,19^ However, their data does show differences in the PROM-specific subscores when assessing the patient data. Regarding differences, Shah et al^20^ demonstrated 5% higher scores of the EuroQoL-D Dimensions (EQ5D) and 14% higher scores of the Visual Analog Scale (VAS) with paper surveys in nonsurgical orthopaedic patients, but 25% higher scores of the Neck Disability Index with tablet-based questionnaires. However, the Bojcic group compared traditional paper and pencil to e-mail surveys for patients who recently underwent ACL reconstruction and showed no differences in PROM scores between mode of administration.^21^

### Postal Surveys

Three articles identified by this meta-analysis examined postal mail's role in PROM acquisition.^21,22,23^ All three studies demonstrated excellent agreement between postal mail and other modes including telephone, in-person interviews, and electronic surveys.

### Telephone Surveys

Our meta-analysis examined four studies that compared scores obtained via phone with that of other methods of data collection. Of the four, three reported no notable differences in scores between the phone and other modalities that included in-person interview, electronic, paper and pencil, and postal.^22,23,24^ One study by Hammarstedt et al^7^ examined modes of PROM acquisition for patients receiving treatment of acetabular labral tears. His group showed that three PROMs (Modified Harris Hip Score [mHHS], Hip Outcome Score – Activities of Daily Living [HOS-ADLS], Hip Outcome Score-Sports Specific Scale [HOS-SSS]) were all notably higher when obtained by telephone versus in-person or online.

### In-person Interview Surveys

The final mode of PROM acquisition is the direct, face-to-face patient interview. Höher et al^25^ examined Lysholm scores at 1-year post-ACL reconstruction obtained by self-administered surveys and direct patient interviews. They found that scores obtained via face-to-face interview were notably higher, by up to 3%, than the self-reported scores.

### Meta-analysis

**Figure 2:**
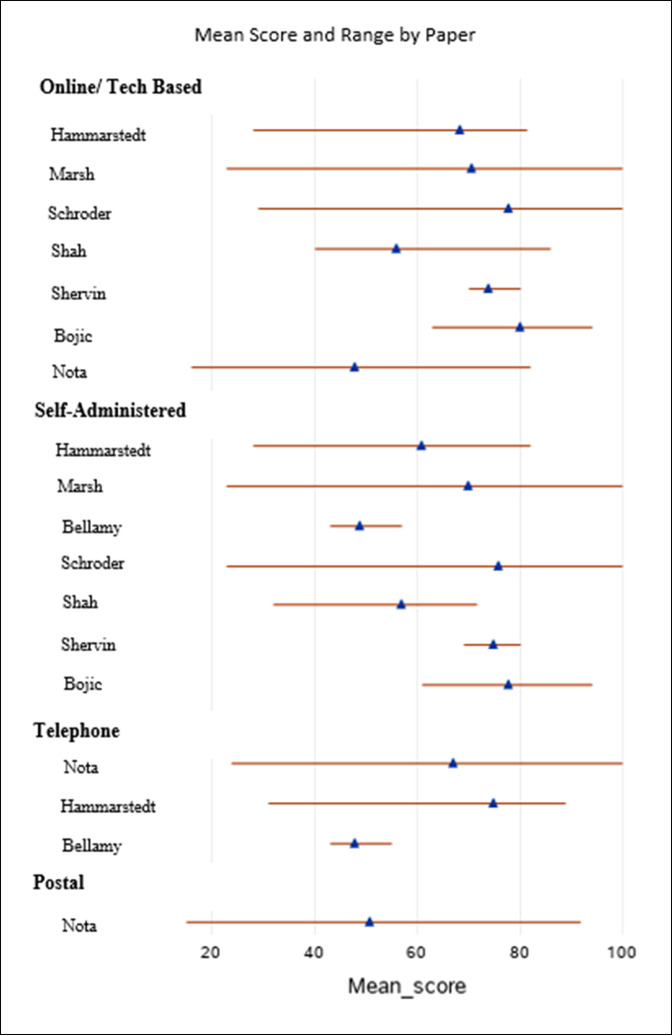
Forest plot demonstrating heterogeneity between studies. Plot is broken down by the mode of survey, and the author represents the article from which the mean scores are derived from. The filled triangle represents the mean score of the patient reported outcome stratified by mode of survey, and the line represents the range of scores reported in the studies.

Mean scores and ranges are visually similar within the mode of surveys (Figure 2). The ranges have some overlap, implying that these data have similar characteristics and scores. The difference in scores can be attributed to the different surgeries, surveys, and sample sizes.

**Figure 3:**
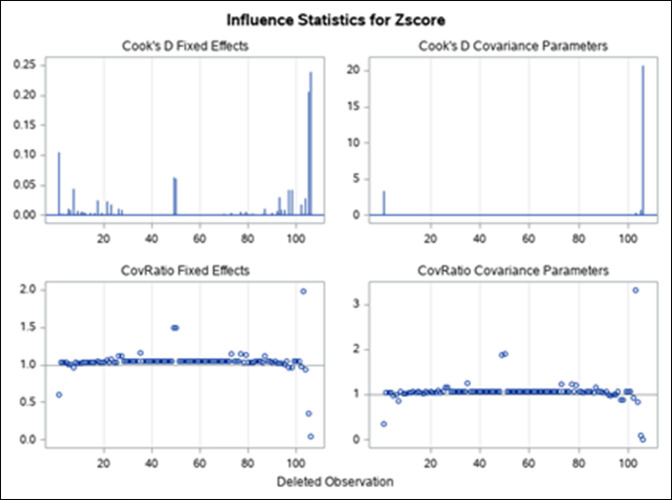
Figure demonstrating the standard panel of influence that is obtained when the mean score analysis is iterative using Cook D and covariance ratio statistic. Cook D measures impact on the estimates when deleted, and the covariance ratio measures the impact on the precision.

The residual maximum likelihood estimate has one observation that displaced the data points. When outliers are removed, a standard panel of influence is obtained when the mean score analysis is iterative using Cook D and covariance ratio statistic that validates the data points used for this meta-analysis (Figure 3). Some mean scores still had considerable impact on the estimates and residuals.

The average/normalized mean effect size for telephone, postal, online/technology based, and in-office self-administered/paper surveys are 71.7, 70.3 (*P* = 0.45), 65.3 (*P* < 0.0001), and 61.8 (*P* < 0.0001), respectively (Table 2). Postal surveys did not have a notable effect size (*P* = 0.45) in comparison to the effect sizes of the other modes of surveys, likely because of power. Telephone surveys have a notably higher effect size, 71.7 (SE 5.0), *P* < 0.0001, compared with online-/tech-based and in-office self-administered/paper survey methods. This indicates that after normalized of scores, PROMs obtained via telephone-based surveys had scores higher than those obtained via the other modalities, up to 8.9% higher than online-/technology-based surveys and 13.8% higher than self-administered/paper surveys.

**Table 2:** Average Effect Size for the Mean Scores for the Different Modes of Surveys Based on Standardized Scale of 0 to 100

Mode of Survey	Effect Estimate (SE)	95% CI	*P*
Telephone	71.7 (5.00)	—	—
Postal	−1.4 (1.90)	-2.3 to 5.1	0.45
Online/tech based	−6.4 (0.70)	5.0 to 7.8	<0.0001
Self-administered paper	−9.9 (0.70)	8.5 to 11	<0.0001

Telephone survey was the reference survey. *P* values generated based on comparisons to telephone effect size. Data demonstrates that telephone scores were notably higher than those obtained via online/technology based or self-administered surveys.

## Discussion

PROMs are a very important and useful tool in the field of orthopaedics. They give providers the information necessary to evaluate treatment efficacy and fuel outcome-driven research that defines clinical and surgical decision-making by allowing comparison between studies. Within orthopaedics, PROMs are the main source for assessing patient's subjective outcomes in the setting of clinical research. However, we do not have an agreed on mode of PROM acquisition. Studies tend to publish data as a cumulative set, rather than properly defining collection methods. In addition, the main goal of researchers within this field is to obtain relevant, reliable patient data with high follow-up percentages. Thus, researchers use multiples modes of delivery to acquire PROM data. It is unclear from our review whether data gathered from differing modes of administration provided a more robust data set with less incomplete data.

This meta-analysis identified several different studies within orthopaedics that examined results of PROMs based on the mode of acquisition. The four main groups examined were electronic-/technology-based surveys, postal surveys, telephone surveys, and in-person interviews. Differing modes of administration are used for several reasons. Researchers may use basic in-person or paper surveys that are easy to complete and tend to not overwhelm patients. However, other researchers use e-mail, phone calls, and other technology-based methods to administer these surveys that can increase the speed of data acquisition, facilitate data integration, and minimize cost. Several studies suggested that notable differences were present in PROM values based on the mode of acquisition, but the delineation of the specific relationship has not yet been made clear. Interview bias has been described in the past, which, in theory, was thought to apply to in-person interviews and telephone-based encounters. This systematic review and meta-analysis is the first, to our knowledge, to directly examine the effects of mode of administration on PROM values within orthopaedics.

Of the 10 included studies in the meta-analysis, the PROMs were normalized to a scale of 0 to 100. This analysis allowed for the comparison of multiple survey types among these different studies. After the normalization process, it was shown that PROMs administered via telephone had a notably higher scores compared with those obtained by both online-/technology-based surveys (8.9% higher) and self-administered/paper surveys (13.8% higher).

Based on findings from this study, we recommend changes in the reporting and publication of orthopaedic studies that use PROMs as a primary outcome measure. Without a full understanding of the degree and magnitude of mode of administration on PROM scores, it is critically important for researchers to strive to use the same mode of administration within studies, and disclose which collection methods are being used for these specific studies. By using the same collection method, researchers can essentially eliminate this potential source of bias within their analyses and allow for comparison across studies without the introduction of a major known confounder. Second, disclosure of collection methodology should become standard practice for readers and reviewers to be aware of the potential introduction of bias. The overall goal was that through further understanding of this collection method bias with orthopaedic surgery, we can use a “correction coefficient” that will allow for standardization of PROMs across different subspecialties and specific surveys.

Limitations to this study exist. First, this study examines multiple different orthopaedic patient populations undergoing different surgeries or clinical evaluation. Second, although every included study was orthopaedic related, the outcomes and PROMs used in each study differed from one another. Although hundreds, and potentially thousands, of studies within the field of orthopaedic surgery use PROMs, very few studies detail their collection methods and even fewer provide adequate statistically data to be included in such a meta-analysis. Thus, underscoring both the difficulty and importance of completing this study. In addition, this study did not account for socioeconomic or demographic data pertaining to the patients that may necessitate variation in the survey administration method. However, for our review, most studies did not disclose the socioeconomic and literacy levels of the patient cohort being studied. Previous studies have shown that these variables may impact access to technology or phones, altering the overall scores reported by the patient population.^15,26^ The timing of survey administration in each study was not consistent, which may represent another confounding variable in the patient data.

As with any meta-analysis, the disadvantage is heterogeneity between study designs, which is controlled for with the random-effects modeling. Data can only be compared through reporting, such as central location, in turn, limiting the number of studies included in the analysis. The including and excluding criteria for the meta-analysis are more stringent; therefore, less control exists on the study designs that are included. The meta-analysis was also performed on this group of studies that used several different PROMs and focused on a wide range of orthopaedic surgeries. Although standardized for comparability in our study, future studies should be done that focus on these mode of acquisition effects for each specific surgery and its respective PROM. Finally, the study design of the articles selected was not homogenous, and thus, a statistical meta-analysis of the data was not standardized to a specific PROM or surgical procedure. In the future, large prospective studies that control for survey timing, mode of administration, and survey type can help to mitigate data inconsistencies and improve accuracy. However, this remains unable to be studied until documentation and reporting of collection methods improve.

Ultimately, this meta-analysis demonstrated differences in PROMs based on the mode of questionnaire administration in the field of orthopaedics. PROM scores obtained via telephone (71.7) are 8.9% higher than scores obtained online (65.3, *P* < 0.0001) and 13.8% higher than scores obtained via self-administered on paper (61.8, *P* < 0.0001). This is the first study that has quantified statistically notable differences between PROM scores based solely on the mode of acquisition across orthopaedic surgery. As PROMs continually become more important to research, clinical and surgical decision-making, and reimbursement, this study can be used to help researchers better understand the confounding effect of mode of acquisition and how to correct for it.
